# Editorial: Contribution of ion channels to neuropathologies

**DOI:** 10.3389/fcell.2023.1179663

**Published:** 2023-03-13

**Authors:** Miklos Kecskes, Steve Peigneur, Katharina Held

**Affiliations:** ^1^ Institute of Physiology, Medical School, University of Pécs, Pécs, Hungary; ^2^ Toxicology and Pharmacology, University of Leuven, Leuven, Belgium; ^3^ Department of Clinical Neurobiology, University Hospital Heidelberg, Medical Faculty of the Heidelberg University and the German Cancer Research Center (DKFZ), Heidelberg, Germany

**Keywords:** ion channels, neuronal disorders, brain, CNS, channelopathies

## Introduction

In our nervous system information is carried in the form of electrical signals that are generated and transduced by the function of ion channels present in the membrane of the neurons (Hille, 2001). Ion channels allow the permeation of ions over the cell membrane, which results in a potential difference between the extracellular space and the intracellular side of the cell. This basic mechanism is accomplished by hundreds of different ligand or voltage-gated ion channels. In fact, ion channel proteins are at the basis of neuronal network activity and a dysregulation of their functions can thus have grievous consequences.

Based on the diversity of ion channels and their unique expression pattern in the different cells within the nervous system, malfunctions of these proteins are described to cause a multitude of different neuronal disorders. Indeed, among the Food and Drug Administration (FDA)-approved drugs, 18% are targeting ion channels (Santos et al., 2016). Decades of studies have shown the pertinent role of ion channels in stirring and sustaining basic cellular and synaptic processes within the neuronal networks of the PNS and CNS. Despite our current knowledge of ion channel contributions to neuronal mechanisms and their subsequent involvement in the development, manifestation and progression of neuropathologies, more research is necessary to decipher the molecular programs that keep our neuronal networks in tune and the role of ion channels therein. Only a detailed understanding of the physiological and pathological functioning of ion channels in our nervous system can provide us with the necessary knowhow in order to develop novel treatment strategies to combat the many severe neuropathologies that are linked to ion channel malfunctions, with the overarching goal to alleviate patient suffering.

This Research Topic includes three original research papers and one review article from prominent researchers in the field and provides insights into recent advances in the field of transient receptor potential (TRP), voltage-gated sodium (Na_V_) and voltage-gated calcium (Ca_V_) channels.

### TRP channels

The TRP multigene superfamily encodes integral membrane proteins that function mainly as sensors for external and internal stimuli (Voets et al., 2005). Most TRP channels are non-selective cation channels, while only a few are Ca^2+^ selective. This channel family shows a variety of gating mechanisms, with modes of activation ranging from ligand binding, to changes in voltage, osmolarity and temperature (Nilius and Owsianik, 2011). They formed the subject of study for two articles in our Research Topic.

The centrally projecting Edinger-Westphal nucleus (EWcp) in the brain is involved in stress adaptation, mood control and energy metabolism by its urocortin 1 (UCN1) expressing peptidergic neurons (Kozicz et al., 2011). Al-Omari et al. discovered that these neurons express functionally active TRPA1 channels that contribute to the physiological functions of EWcp neurons including the regulation of alcohol consumption. Specifically, they showed that alcohol treatment decreased *Trpa1* mRNA expression as well as UCN1 peptide content while it did not affect the *urocortin 1* mRNA expression in EWcp neurons. Consequently, their result suggests that TRPA1 signaling may contribute to both the storage and release of UCN1 peptide. Further research is needed to clarify how exactly TRPA1 activation leads to UCN1 release.

Konkoly et al. studied the same group of neurons in the EWcp nucleus in experimental posttraumatic stress disorder (PTSD). They found decreased *Trpa1* mRNA expression after stress exposition but increased UCN1 content in EWcp neurons. Furthermore, stressed *Trpa1* ^*−/−*^ mice displayed reduced immobility in forced swim and restraint tests compared to stressed wild type mice. These results suggest that TRPA1 might be involved in the regulation of stress adaptation by modulating UCN1-positive neurons in the EWcp.

### Voltage-gated ion channels

Voltage-gated ion channels are a class of transmembrane proteins that are strongly activated by changes in the membrane potential in the proximity of the channel. They are often specific to one particular ion and therefore selectively permeable to sodium (Na_V_ channels), potassium (K_V_ channels), calcium (Ca_V_ channels) or chloride (CLC channels) ions (Purves et al., 2018). In this Research Topic, the roles of Na_V_ and Ca_V_ channels in neuropathologies are investigated and reviewed.

A SCN1B mutation (c.308A>T) leading to a D103V amino acid alteration in the Na_V_β1 and Na_V_β1b subunits was recently identified in a patient with cardiac, cognitive and motor deficits as well as brain abnormalities (Eldomery et al., 2017). Na_V_β subunits are described as auxiliary subunits controlling the biophysical properties of the ion channel pores formed by Na_V_α subunits (Hille, 2001). Martinez-Moreno et al. could illuminate that Na_V_β1^D103V^ induces a loss of function in cardiac (Na_V_1.5) as well as brain (Na_V_1.1) sodium channels due to a reduction in current densities. Oppositely, Na_V_β1b^D103V^, which is predominantly expressed during early developmental stages, induced a Na_V_1.1 gain-of-channel function mediated by a rightward shift of the voltage dependence of inactivation and a faster recovery from inactivation. Altogether, both loss and gain of Nav1.1 function at different developmental stages may contribute to the neurological deficits and brain abnormalities of the patient.

Antunes et al. provide an overview on how a subclass of Ca_V_ channels in presynaptic terminals, namely, the N-type Ca^2+^ channels (NTCCs), present viable targets for the treatment of neurological disorders such as Huntington’s disease, multiple sclerosis, and migraine, which have been shown to all share NTCCs regulated pathways. In particular, the use of NTCC blockers (often peptides derived from animal venoms) and their potential to counter demyelination, neuroinflammation, pain and neurotransmitter release in these neurological conditions is reviewed and discussed.

## Conclusion

This Research Topic provides novel scientific advances and deeper insights into the role and regulations of ligand and voltage-gated ion channel functions in neurological disorders, covering research on three different channel families in neurological diseases ranging from addiction, over stress and pain to developmental, cognitive, and neurodegenerative disorders. Besides the new knowledge that was obtained within this Research Topic, also novel research questions arose that carry great scientific merit to be explored in the near future.
